# Immunogenicity and Protective Activity of Pigeon Circovirus Recombinant Capsid Protein Virus-Like Particles (PiCV rCap-VLPs) in Pigeons (*Columba livia*) Experimentally Infected with PiCV

**DOI:** 10.3390/vaccines9020098

**Published:** 2021-01-28

**Authors:** Huai-Ying Huang, Benji Brayan I. Silva, Shen-Pang Tsai, Ching-Yi Tsai, Yu-Chang Tyan, Tzu-Che Lin, Ronilo Jose D. Flores, Kuo-Pin Chuang

**Affiliations:** 1 International Degree Program in Animal Vaccine Technology, International College, National Pingtung University of Science and Technology, Pingtung 912, Taiwan; vetlaenwe@gmail.com (H.-Y.H.); ksrobert@ms24.hinet.net (S.-P.T.); j10785002@g4e.npust.edu.tw (C.-Y.T.); 2 Graduate School, University of the Philippines Los Baños, Laguna 4031, Philippines; bisilva@up.edu.ph (B.B.I.S.); rdflores3@up.edu.ph (R.J.D.F.); 3 Graduate Institute of Animal Vaccine Technology, College of Veterinary Medicine, National Pingtung University of Science and Technology, Pingtung 912, Taiwan; yctyan@kmu.edu.tw; 4 School of Medicine, College of Medicine, Kaohsiung Medical University, Kaohsiung 807, Taiwan; 5 Department of Medical Imaging and Radiological Sciences, Kaohsiung Medical University, Kaohsiung 807, Taiwan; 6 Institute of Medical Science and Technology, National Sun Yat-sen University, Kaohsiung 804, Taiwan; 7 Graduate Institute of Medicine, College of Medicine, Kaohsiung Medical University, Kaohsiung 807, Taiwan; 8 Department of Medical Research, Kaohsiung Medical University Hospital, Kaohsiung 807, Taiwan; 9 Center for Cancer Research, Kaohsiung Medical University, Kaohsiung 807, Taiwan; 10 Department of Plant Industry, College of Agriculture, National Pingtung University of Science and Technology, Pingtung 912, Taiwan; tclin@mail.npust.edu.tw; 11 Institute of Biological Sciences, College of Arts and Sciences, University of the Philippines Los Baños, Laguna 4031, Philippines; 12 Research Center for Animal Biologics, National Pingtung University of Science and Technology, Pingtung 912, Taiwan; 13 School of Dentistry, Kaohsiung Medical University, Kaohsiung 807, Taiwan

**Keywords:** pigeon circovirus (PiCV), young pigeon disease syndrome (YPDS), virus-like particles (VLPs), immunogenicity, mammalian expression system

## Abstract

Pigeon circovirus (PiCV) is the most recurrent virus diagnosed in pigeons and is among the major causative agents of young pigeon disease syndrome (YPDS). Due to the lack of an established laboratory protocol for PiCV cultivation, development of prophylaxis is hampered. Alternatively, virus-like particles (VLPs), which closely resemble native viruses but lack the viral genetic material, can be generated using a wide range of expression systems and are shown to have strong immunogenicity. Therefore, the use of VLPs provides a promising prospect for vaccine development. In this study, transfected human embryonic kidney (HEK-293) cells, a mammalian expression system, were used to express the PiCV capsid protein (Cap), which is a major component of PiCV and believed to contain antibody epitopes, to obtain self-assembled VLPs. The VLPs were observed to have a spherical morphology with diameters ranging from 12 to 26 nm. Subcutaneous immunization of pigeons with 100 µg PiCV rCap-VLPs supplemented with water-in-oil-in-water (W/O/W) adjuvant induced specific antibodies against PiCV. Observations of the cytokine expression and T-cell proliferation levels in spleen samples showed significantly higher T-cell proliferation and IFN- γ expression in pigeons immunized with VLPs compared to the controls (*p* < 0.05). Experimentally infected pigeons that were vaccinated with VLPs also showed no detectable viral titer. The results of the current study demonstrated the potential use of PiCV rCap-VLPs as an effective vaccine candidate against PiCV.

## 1. Introduction

The pigeon circovirus (PiCV) is among the major causative agents of a multifactorial disease known as the young pigeon disease syndrome (YPDS) [1]. PiCV genome is composed of a single-stranded circular DNA with a length of approximately 2000 nucleotides. The PiCV genome is characterized by two major open reading frames (ORFs), ORF C1 and ORF V1.

ORF C1 encodes a 30 kDa protein that is responsible for the assembly of the viral capsid [2]. On the other hand, ORF V1 encodes a nonstructural protein, the replication-associated protein (Rep) [3]. A canonical nonanucleotide motif (NANTATTAC) at the peak of a stem-loop located between the 5′-ends of the two main ORFs (C1 and V1), which is considered to be a requirement for the initiation of the viral genome replication, is also present [4]. 

For more than two decades, one of the common problems in young racing pigeons is YPDS, which is associated with poor racing performance, and high morbidity and mortality rates among pigeons of age 3 to 20 weeks [5]. The strong immunosuppressive capacity of PiCV infections increases the probability of secondary infections that result in huge losses [6]. Infected birds display nonspecific symptoms, such as anorexia, lethargy, poor racing performance, depression, diarrhea, a fluid-filled crop [7], polyuria, rapid weight loss, ruffled feathers, and vomiting [8,9,10].

The capsid protein of circoviruses is believed to display antigenic properties that induce antibodies when the host is infected with the virus. This was verified in the cases of beak and feather disease virus (BFDV), PiCV, and porcine circovirus genotype 2 (PCV2) [11,12,13]. An approved commercial vaccine or prophylaxis against PiCV is not yet available, since there is no laboratory protocol for culturing the virus [14,15]. To date, detection of PiCV infection involves the use of conventional methods including histological observation, electron microscopy, and molecular diagnosis, such as in situ hybridization, nucleic acid-based dot blot hybridization and polymerase chain reaction (PCR) [16,17,18].

Virus-like particles (VLPs) are multiprotein structures that mimic the characteristics of the virus, which are ideal for vaccine development [19]. VLPs lack the viral genome necessary for viral replication, making them one of the safest templates for vaccine development. VLPs can assemble into different conformations, but generally tend to assemble into viral-like structures that range in diameters of 25–100 nm [20]. The viral morphology of VLPs brought advantages to their immunostimulatory activity because it can: (1) be efficiently recognized by antigen presenting cells; (2) be trafficked from the site of injection to the lymph nodes; and (3) be a promoter of B-cell activation due to its repetitively arranged structural features, which result in a stronger humoral immune response, along with cellular mediated immunity and enhanced T-cell stimulation [21]. The highly ordered structure and multivalent display of VLPs may also facilitate recognition by pathogen-associated molecular pattern motifs that can trigger innate immune sensing mechanisms and can be recognized by Toll-like receptors and other pattern-recognition receptors that are present in host cells [22,23]. As of today, only one study has been performed using VLPs as a potential vaccine for PiCV, and they found that VLPs induce antibody response in immunized mice. However, in this study, a baculovirus expression system was used, but no challenge test was conducted; thus, a comparison of the viral titers of challenged vaccinated and nonvaccinated mice was also not reported [24]. With a combination of strong immunogenicity and good safety profiles, VLPs are expected to acquire widespread recognition in different fields, such as vaccines, therapeutic modalities, and in vitro diagnostics [25].

Due to the high prevalence of PiCV and with no available vaccines, severe losses were incurred by the poultry and pigeon racing industry. Because of the persistent threats posed by PiCV, vaccine development would be of great interest. 

In this study, recombinant capsid protein was generated using a mammalian expression system. Transfected human embryonic kidney (HEK-293) cells were used to produce self-assembled VLPs. Furthermore, challenge tests and qPCR analysis of cytokines were done to determine the immune response post-immunization. The current study is limited to the development of a vaccine against PiCV alone.

## 2. Materials and Methods

### 2.1. Cell Line

Human embryonic kidney 293 cells (HEK-293) (ATCC^®^ CRL-1573™) were cultured and propagated in Dulbecco’s Modified Eagle Medium (DMEM) (Gibco, Gaithersburg, MD, USA) supplemented with 10% fetal bovine serum (FBS) (Hyclone laboratories, Inc., Logan, UT, USA) and with 50 µg/mL of penicillin-streptomycin (Gibco, USA) and incubated at 37 °C and 5% CO_2_.

### 2.2. Construction of Recombinant Capsid Protein (rCap) for Escherichia coli and Mammalian Cell Expression System

For the *E. coli* expression of the rCap, the *cap* gene sequence of PiCV P99/05 strain (GenBank Accession No. HQ401274.1) was codon usage-optimized by Genomics, New Taipei City, Taiwan and synthesized in pGS21-a (GenScript Biotech Corp., Piscataway, NJ, USA) with EcoRI and XhoI as restriction sites. The constructed plasmid contains a genomic sequence that encodes PiCV capsid gene with a C-terminal GST tag and an N-terminal His-tag. On the other hand, for the mammalian cell expression system, the same gene sequence (GenBank Accession No. HQ401274.1) was codon usage-optimized (Genomics, New Taipei City, Taiwan) and synthesized in pCDNA3.1 (Protech Technology Enterprise Co., Ltd., Taipei, Taiwan) with KpnI and NotI as restriction sites. The constructed plasmid contains a genomic sequence that encodes PiCV capsid gene with His-tag. Verification of the constructed sequence was performed through DNA sequencing. The resulting plasmid was used for transfection and protein expression experiments.

### 2.3. E. coli Expression of rCap

One liter (1 L) of LB was inoculated with *E. coli* (BL21) containing the recombinant pGS21a-Cap plasmid and incubated at 37 °C until O.D. 600 reached 0.6. Afterward, rCap expression was induced by adding 1 mM Isopropyl β-D-1-thiogalactopyranoside (IPTG) to the medium and subsequently incubating for another 6 h at 37 °C. After incubation, the culture was then centrifuged at 10,000 rpm for 10 min. The resulting bacterial pellet was resuspended in 10 mL Tris-buffered saline (TBS), and the mixture was sonicated thrice in 20% pulsing cycles (MISONIX Sonicator^®^ 300, Sunwise, Taipei, Taiwan). Cell debris from the sonication was removed by centrifugation at 10,000 rpm for 10 min. The supernatant was subjected to Ni2+ affinity column chromatography to purify the collected recombinant capsid proteins. Purity of the obtained rCap was assessed by SDS-PAGE Analysis and Western blot analysis. The purified rCap was then stored at −80 °C for further use.

Briefly, 10 μL of protein samples was separated using 12% of SDS-PAGE and was visualized by Coomassie blue dye. For Western blot analysis, separated proteins were electrophoretically transferred onto polyvinylidene difluoride (PVDF) membrane. Unwanted sites on the membrane were blocked by incubating in 10 mL of 5% skim milk (Anchor, New Young, Singapore, Singapore) with shaking for 1 h at 37 °C. The membrane was washed with PBST (0.1% Tween 20 in 1x PBS) 5 times for 5 min each. After washing, the membrane was incubated with mouse anti-His primary antibody (TOOls, Taipei, Taiwan) diluted 3000-fold in 10 mL 5% skim milk with shaking at 4 °C for 12 h to enable the binding of primary antibody with the histidine tag (6x His-tag) of the target protein. After the addition of primary antibody, the membrane was again washed with PBST 5 times for 5 min each. Next, the membrane was incubated with horse radish peroxidase (HRP)-conjugated rabbit anti-mouse IgG (ZYMED, South San Francisco, CA, USA) diluted 5000-fold in 10 mL 5% skim milk with shaking for 1 h at 37 °C. Using a chemiluminescent substrate for the detection of HRP (West Pico PLUS Chemiluminescent Substrate, Thermo, Waltham, MA, USA), the blot was visualized by X-ray film development.

### 2.4. Precipitation of Virus-Like Particles (VLPs)

The pCDNA3.1-Cap vector was transfected into HEK-293 cells using lipofectamine (Invitrogen, Carlsbad, CA, USA) to produce PiCV VLPs. The medium containing the cells was collected after 72 h of incubation at 37 °C, and it was subjected to centrifugation at 1500 rpm for 5 min to separate the cells from the medium. The cells were washed using PBS, and the mixture was centrifuged again for another 5 min at 1500 rpm. Collected cells were mixed with whole cell extract buffer and then placed on ice for about 30 min. Afterward, the mixture was centrifuged at 10,000 rpm for 10 min. The supernatant was precipitated by adding 7% poly-ethylene glycol (PEG) and 2% NaCl solution, and then agitating it at 4 °C for 4 h. After agitation, it was centrifuged with a speed of 9500 rpm at 4 °C for 30 min. The supernatant was disposed, while the pellet containing the PiCV VLPs was resuspended in 4 mL PBS. The resulting VLPs solution was stored in −20 °C for further characterization, and until use for immunization. SDS-PAGE and subsequent Western blotting were performed as described above, but the blot was visualized using a digital imaging system (Fusion Solo S, VILBER LOURMAT, Eberhardzell, Germany). For size comparison, the protein weight marker captured under visible light was automatically merged with the image of the blot by the imaging program. Electron microscopy was also performed to observe the morphology of the VLPs.

### 2.5. Electron Microscopy

The purified PiCV rCap-VLPs were captured on a carbon coated slotted grid, which was then stained with phosphotungstic acid (PTA) for 60 s. After staining, a transmission electron microscope (TEM) (H-7500, Hitachi, Tokyo, Japan) was used to visualize the morphology of the VLPs.

### 2.6. Pigeons and Ethics Statement

Twenty (20) twenty-one (21) day old pigeons (*Columba livia*) obtained from a private hatchery were used in this study. Prior to immunization, the antibody titers of pigeons against PiCV capsid protein were measured by ELISA at 21 and 28 days of age. Pigeons with serum ELISA optical densities (O.D.) higher than that of the 0 day old PiCV-free pigeon serum (negative control) were not selected for use in this study. The pigeons selected for both the control and VLP groups were subcutaneously (S.C.) immunized twice at age 28 and 42 days. The control group was immunized with saline solution supplemented with water-in-oil-in-water (W/O/W) adjuvant (Summit P-168, Country best biotech, Taipei, Taiwan), while the VLP group was immunized with 100 μg PiCV rCap-VLPs supplemented with W/O/W adjuvant. On 0, 14, and 21 days post-vaccination (dpv), blood samples were collected from the wing vein of 5 pigeons in each group to measure the antibody titer. Spleen samples were also collected from 5 pigeons from each group at 21 dpv for T-cell proliferation and cytokine assays. Additionally, at 21 dpv, a challenge test was administered to 10 pigeons (5 pigeons per group) by giving the pigeons feed containing 3 g of lymphoid tissues containing 6 × 10^3.5^ copies/g of PiCV. All pigeons were euthanized using CO_2_ at 28 dpv for histopathological examination and quantification of the viral load.

All animal tests were conducted as stated in the ethical guidelines for animal rights protection of the National Pingtung University of Science and Technology-Institutional Animal Care and Use Committee (NPUST-IACUC), with IACUC permit number NPUST 107-056.

### 2.7. Sample Collection, Fixation, and Histopathological Examination

Following the euthanasia of pigeons, tissue samples from the spleen were collected. The extracted tissue samples were fixed in 10% phosphate buffer formalin solution and embedded in paraffin. For histopathological examination, hematoxylin-eosin was used to stain 5 µm thick tissue samples. The samples were examined under a light microscope, and images were obtained using a Nikon DS-L2 camera unit connected to a Nikon Eclipse E-200 microscope. 

### 2.8. Indirect Enzyme-Linked Immunosorbent Assay

To determine the PiCV-specific IgG antibodies, the collected sera from the wing vein of the pigeons were each tested by ELISA. Coating buffer (Candor Bioscience GmbH, Wangen im Allgäu, Germany) containing 40 μg/mL of purified capsid protein expressed in *E. coli* was used to coat the wells of the ELISA plate. Each of the collected pigeon sera were diluted 120-fold in the assay buffer, and 100 μL of the serum was deposited in each well. Serum from 0 day old PiCV-free pigeons, diluted 512-fold, was used as negative control. For the secondary antibody, horseradish peroxidase (HRP)-labeled goat anti-pigeon IgG (OrigGene Technologies, Rockville, MD, USA) diluted to 6000-fold was used. The assay was carried out with ABTS (2,2′-azino-bis(3-ethylbenzothiazoline-6-sulphonic acid)) substrate for 15 min. An ELISA plate reader was used to read the optical density (OD) at 405 nm.

### 2.9. T-Cell Proliferation Analysis

An MTT (3-(4,5-dimethylthiazol-2-yl)-2,5-diphenyltetrazolium bromide) assay was used to determine the T cell proliferation activity in the spleen samples. Spleen samples were macerated through a sieve using a syringe plunger to obtain single-cell suspension in Hank’s Balanced Salt Solution (HBSS). The cell suspensions were overlaid onto Histopaque^®^ 1077 density gradient medium and centrifuged at 1800 rpm for 20 min at room temperature. Lymphocytes at the interface were collected and washed three times in HBSS. Lymphocytes isolated from the spleen of both groups were seeded into a 96-well plate. Each well was seeded with 1 × 10^5^ cells in 100 μL medium (10% FBS DMEM with penicillin and streptomycin). The cells were then incubated with 10 μg of VLPs for 72 h at 37 °C. Following the incubation, 10 μL of MTT with a concentration of 0.5 mg/mL was added into each well, and the plate was further incubated for another 4 h at room temperature. The medium inside the ELISA plate was collected and centrifuged at 1500 rpm for 15 min. Following centrifugation, the supernatant was discarded, and the cells were lysed in a buffer (0.01 M HCl + 10% SDS). An ELISA reader (Bio-TEK Instruments, Inc., Winooski, VT, USA) was used to measure the extent of color change at 570 nm. T cell proliferation activity was expressed as stimulation index (S.I.).

### 2.10. Cytokine Analysis by RT-PCR

Lasergene package (DNAStar Inc., Madison, WI, USA) was used to design a RT-PCR primer pair specific for IFN-γ, TGF-β, and IL-8. Pigeon β-actin was used as internal control (Table 1). Amounts of 500 mg of spleen tissue samples (frozen and thawed three times) were first digested with 1 mL TRIzol (Invitrogen, Taipei, Taiwan) and vortexed for 15 s. Then, the samples were incubated at room temperature for 15 min followed by centrifugation at 12,000 rpm for 15 min to separate the nucleic acid and protein. The aqueous phase was collected and transferred to a clean tube, and 750 μL absolute isopropanol was added. After gently inverting the tube several times, it was placed at −20 °C for 20 min. The solution was centrifuged at 12,000 rpm for 10 min to isolate the mRNA. The supernatant was removed, and 500 μL of 75% isopropanol solution in diethyl-pyrocarbonate-treated water (DEPC H_2_O) was added to wash out excess salts followed by centrifugation at 8000× *g* for 2 min. Excess alcohol was drained, and the RNA pellets were air-dried for 30 min and re-suspended in 50 μL of DEPC H_2_O. RNA concentration was measured using OD at 260 nm, and its quality was evaluated by calculating the OD260/OD280 ratio. Complementary DNA was produced from 100 to 200 ng of RNA per reaction. The RNA was subjected to RT-PCR using a RevertAid First Strand cDNA Synthesis Kit (ThermoFisher, Waltham, MA, USA) following the manufacturer’s instructions. Furthermore, RT-PCR was carried out using a thermal cycler (Applied Biosystems™, Thermo, Waltham, MA, USA) with the following conditions: initial denaturation at 95 °C for 3 min, 40 cycles of denaturation at 95 °C for 30 s, annealing at 60 °C for 30 s, and elongation at 72 °C for 30 s. The assay was performed using the StepOne™ Real-Time PCR System (Applied Biosystems™, Thermo, USA). The calibrated cytokine content relative to the control group was evaluated through the comparative Ct method (2^−∆∆^Ct).

### 2.11. Detection of Viral Load Using qPCR

To detect the vial load, qPCR primers used were designed specifically for PiCV and pigeon β-actin (Table 1) using Lasergene package (DNAStar Inc., Madison, WI, USA). The primers were synthesized by Genomics (Genomics, Taipei, Taiwan). The extracted DNA from spleen and liver were mixed with the qPCR mixture containing the designed primer, qPCRBIO SyGreen Blue Mix Hi-ROX (PCRBIOSYSTEM, London, UK), and nuclease free water. qPCR was carried out using the following conditions: initial denaturation at 95 °C for 3 min, 40 cycles of denaturation at 95 °C for 30 s, annealing at 60 °C for 30 s, and elongation at 72 °C for 30 s. The assay was performed using the StepOne™ Real-Time PCR System (Applied Biosystems™, Thermo, USA). Similar to the cytokine analysis, normalized virus titer relative to the control group was also evaluated through the comparative Ct method (2^−∆∆^Ct).

### 2.12. Statistical Analysis

GraphPad Prism 5 (GraphPad, San Diego, CA, USA) was used to carry out all statistical analyses in the study. A two-tailed Student’s t-test was used to conduct statistical comparisons between the control and VLP groups setting the significance level at *p* < 0.05. All data are shown as the mean ± standard deviation [26].

## 3. Results

### 3.1. Protein Expression and Morphological Analysis of Virus-Like Particles (VLPs)

**Figure 1:**
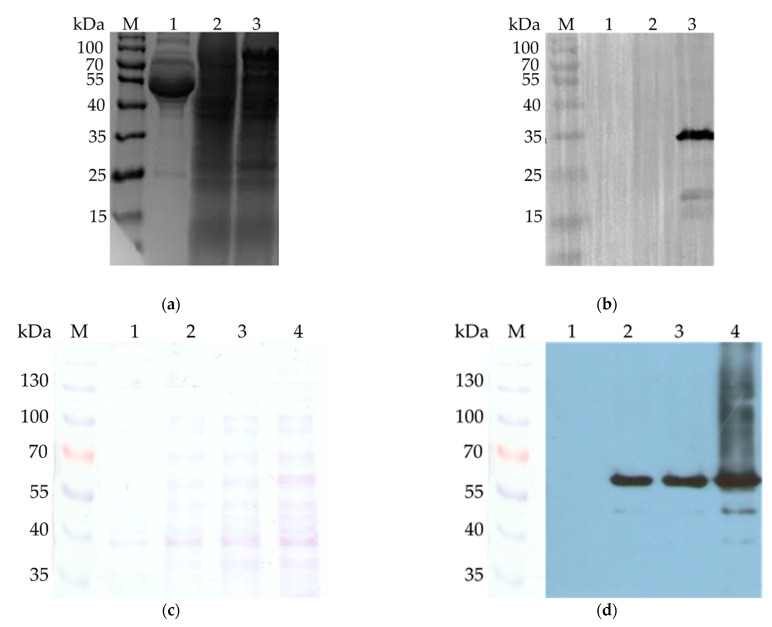
Prokaryotic and mammalian cell expression of PiCV capsid protein. (**a**) SDS-PAGE analysis of PiCV rCap-virus-like particle (VLP) expression in HEK-293 cells. Lane M shows the protein marker (10–170 kDa). Lanes 1, 2, and 3 show the visualized proteins isolated from the culture medium of the transfected cell, cell lysates of the nontransfected and transfected cells, respectively. (**b**) Western blotting analysis of the protein samples using mouse anti-His antibody and horseradish peroxidase (HRP)-conjugated rabbit anti-mouse IgG. Lanes contain similar samples as (**a**). (**c**) SDS-PAGE analysis of PiCV rCap expression in *E. coli* cells. Lane M shows the protein marker (10–170 kDa). Lanes 1, 2, 3, and 4 show the visualized proteins isolated from the lysate of non-Isopropyl β-D-1-thiogalactopyranoside (IPTG)-induced cells, and lysates of 1 h-, 2 h- and 3 h-IPTG-induced cells, respectively. (**d**) Western blotting analysis of the protein samples using mouse anti-His antibody and horseradish peroxidase (HRP)-conjugated rabbit anti-mouse IgG. Lanes contain similar samples as (**c**).

**Figure 2:**
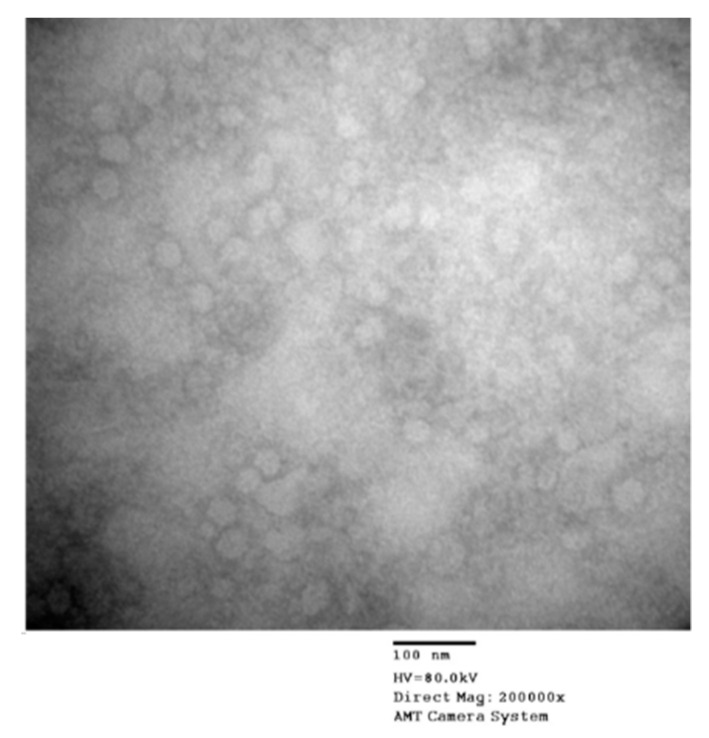
Morphology of virus-like particles (VLPs) as shown in transmission electron microscopy (TEM). Drawn to scale: 100 nm. Detected protein as seen in Figure 1b was purified by PEG precipitation using 7% PEG 6000 solution and 2% NaCl solution to verify presence of PiCV VLPs. Purified VLPs were used to coat a slotted carbon grid and stained with phosphotungstic acid (PTA) before viewing under transmission electron microscope (TEM).

The recombinant expression plasmid pCDNA3.1 was assembled for an effective expression of the target proteins in mammalian cell. Constructed pigeon circovirus recombinant capsid protein virus-like particles (PiCV rCap-VLPs) were successfully acquired by transfection of HEK-293 cells. SDS-PAGE analysis of the expressed capsid protein was shown in (Figure 1a). Western blot showed a 35 kDa band that corresponds to the PiCV rCap verified the presence of the protein. Results from this assay showed that the expressed PiCV rCap was in the cell lysates and not released into the medium (Figure 1b). To further confirm that the Cap protein formed VLPs, electron microscopy was conducted to reveal the morphology of PiCV VLPs. It was observed that the diameters of VLPs were approximately 12 to 26 nm (Figure 2).

Furthermore, PiCV capsid protein was expressed and induced in *E. coli* (BL21) cells using 1 mM IPTG. Successful expression was observed using SDS-PAGE (Figure 1c), and further analysis was performed by Western blot (Figure 1d). Lane 1 represents the cells before IPTG induction, while lanes 2 to 4 represent the cells which were induced with IPTG for 1, 2, and 3 h, respectively, to express the protein. SDS-PAGE analysis showed that the purified rCap has a purity of greater than 95%. Accounting for the 26 kDa size of the GST-tag in addition to the 35 kDa size of the PiCV capsid protein, the Western blot showed an approximately 60 kDa band.

### 3.2. Antibody Titer in Pigeons Post-Immunization with PiCV rCap-VLPs

**Figure 3:**
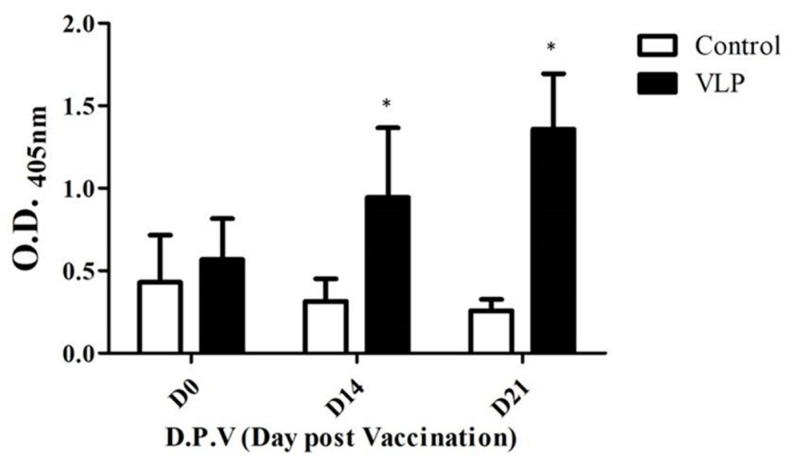
Antibody response of pigeons to PiCV rCap-VLPs vaccination. Two groups (10 pigeons/group) were utilized in this study. The control group was immunized with saline supplemented with water-in-oil-in-water (W/O/W) adjuvant, while the VLP group was immunized with PiCV rCap-VLPs. Pigeons in both groups were immunized twice (at 28 and 42 days of age). Antibody titers were determined at 0, 14, and 21 days post-vaccination (dpv) by ELISA quantified by optical density reading at 405 nm. Significant difference between groups (*p* < 0.05) was notated as *.

Antibody titers of the pigeons were measured on 0, 14, and 21 days post-vaccination (dpv) using ELISA. As shown in (Figure 3), the measurements of antibody titers were in terms of optical density (O.D.) at 405 nm. At 0 dpv, the antibody titer between the control and immunized group did not have any significant differences. However, at 14 dpv, it was observed that the antibody response of the VLP group was significantly higher than that of the control group (*p* < 0.05). This trend continued to increase as observed at 21 dpv when the antibody titer of the VLP group reached an ELISA titer O.D. 405 value of approximately 1.4 (*p* < 0.05). Overall results verified the effectiveness of VLPs in inducing antibody response.

### 3.3. Virus Titer in Pigeons Challenged with Pigeon Circovirus (PiCV)

**Figure 4:**
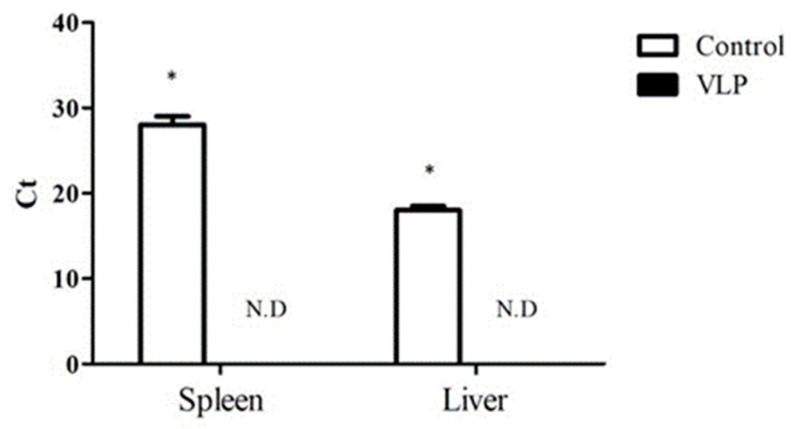
Viral load in the spleen and liver of pigeons. Pigeons from the control group and the PiCV rCap-VLPs immunized were challenged with lymphoid tissues that have 6 × 10^3.5^ copies/g of PiCV. Following immunization and challenge, viral loads in the lymphoid organs of the pigeons were quantified by qPCR and the data are presented as Ct. Significant difference (*p* < 0.05) was notated as *. ND means not detected.

In addition to the evaluation of antibody response to VLPs, viral load in each group was measured in relation to Ct values (Figure 4). The viral titer was measured using spleen and liver samples from the control and VLP group after oral administration of 3 g lymphoid tissues that have 6 × 10^3.5^ copies/g of PiCV. As shown in Figure 4, both the spleen and liver from the control group have significantly higher Ct values as compared to the VLP group (*p* < 0.05). Moreover, Ct values were undetected for both spleen and liver of the VLP group, which signified that the pigeons immunized with VLPs showed no detectable levels of PiCV (*p* < 0.05).

### 3.4. T-Cell Proliferation and Cytokine-Quantities Post-Vaccination

**Figure 5:**
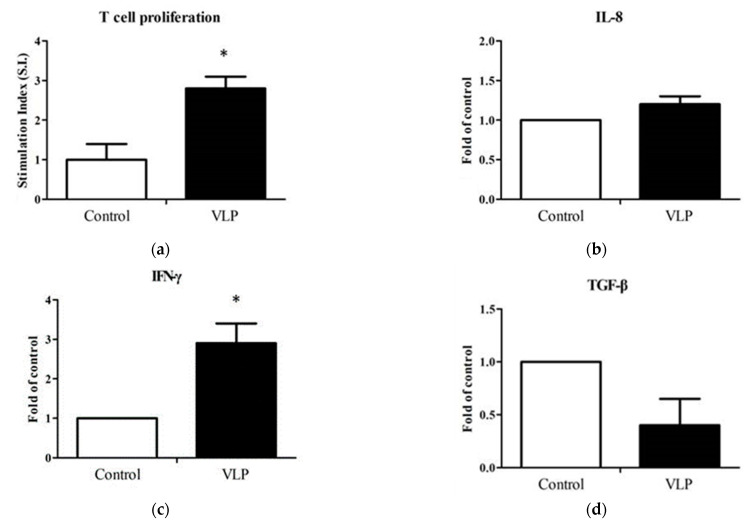
Immunogenic response of pigeons to PiCV rCap-VLPs vaccination. Two groups were observed in the study. The control group was immunized with saline supplemented with W/O/W adjuvant, while the VLP group was immunized with PiCV rCap-VLPs. (**a**) Proliferation of T-cells was determined by isolating lymphocytes from spleen, and subsequently incubating with VLPs protein for 72 h. Quantifications of the expression of each cytokines (**b**) IL-8, (**c**) IFN-γ, and (**d**) TGF-β-2 were performed using RT-PCR, with pigeon β-actin as internal control. Significant difference (*p* < 0.05) was notated as *.

To further investigate the immune response of pigeons vaccinated with rCap-VLP, T-cell proliferation and cytokine expression profiles were evaluated as presented in Figure 5. The expression of IL-8, IFN-γ, TGF-β2, as well as T cell proliferation were observed after the pigeons were subcutaneously administered with rCap-VLP. T cell proliferation in VLP group was found to be significantly higher than the control group (Figure 5) and it reached a stimulation index (S.I.) of 2.8-fold (*p* < 0.05).

Expression of IFN-γ of the immunized group was significantly upregulated with a fold change of 3-fold, which is higher than the control (*p* < 0.05). However, fold change of IL-8 in the spleen samples of both the control and immunized group did not differ significantly.

Additionally, the fold change of TGF-β-2 in the spleen of the VLP group decreased significantly as compared to the control group. It was observed that the expression of TGF-β2 in the VLP group was approximately 0.25-fold, a value that is much less in comparison to that of the control group (*p* < 0.05). This result signified that in this experiment, immunization with rCap-VLP resulted to a suppression of TGF-β-2 expression in the spleen sample.

### 3.5. Histopathological Examination through Hematoxylin-Eosin Staining of Spleen Samples

**Figure 6:**
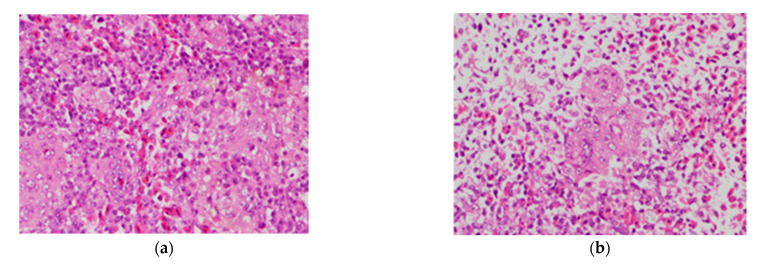
Histopathological examination of spleen samples. Spleen sample from challenged pigeons that were (**a**) immunized and (**b**) nonimmunized with PiCV rCap-VLPs. Spleen samples were stained with hematoxylin-eosin (H&E) stain and were observed under light microscope.

To further confirm the efficacy of PiCV rCap-VLPs as potential vaccines, spleen samples from pigeons that were orally administered with feeds containing PiCV-positive lymphoid tissues were collected and subjected to histopathological examination using hematoxylin-eosin (H&E) staining. Results showed that immunization with PiCV rCap-VLPs helped maintain the integrity of lymphocytes in the spleen (Figure 6a). In contrast, the nonimmunized group revealed a significant decrease in the color of hematoxylin-eosin (H&E) stain, signifying the occurrence of lymphopenia (Figure 6b). These results confirmed the effectiveness of immunization with PiCV rCap-VLPs in regulating the copies of PiCV.

## 4. Discussion

Currently, there are no available prophylaxis against Young Pigeon’s Disease (YPDS), resulting in severe losses to pigeon meat and racing industries [24]. Information on the pathogenesis of the virus and laboratory protocols for culturing pigeon circovirus (PiCV) is also not available. Given the high prevalence of the occurrence of PiCV in pigeon flocks, future development of effective vaccines against this virus could be a possible approach to the treatment of YPDS, considering that this has been conducted in the case of porcine circovirus [12,27,28,29]. In recent years, the production of recombinant capsid protein of various circoviruses, such as duck circovirus (DuCV) [30], porcine circovirus type 2 (PCV2) [31], beak and feather disease virus (BFDV) [11], and PiCV, produced in bacterial, yeast, and baculovirus systems, has already been reported. For this reason, several experiments that assessed the immunogenicity of PiCV rCap in pigeons were designed [13]. Considering the literature mentioned above, insights from this study can aid future development efforts to produce an effective vaccine against PiCV.

Virus-like particles (VLPs) are self-assembled macromolecules that mimic the viral protein structure of a target virus but contain no genetic materials of the native viral strain. VLPs exhibit antigenic epitopes that are positioned in a correct conformation and in a highly repetitive manner [32]. Hence, VLPs have the advantages of both the whole-virus vaccines and recombinant subunit vaccines, which have increased stability, preservation of native antigenic confirmation, and sufficient production. To date, there are several prokaryotic and eukaryotic systems, such as yeast, insect, plant, *E. coli*, and mammalian cells, that have been used to express recombinant proteins for the generation of VLPs. Among these expression systems, eukaryotic expression hosts, such as yeast (*Saccharomyces cerevisiae*, *Pichia pastoris*, and *Hansenula polymorpha*) and mammalian cells (Chinese hamster ovary cell line [CHO]), are used for generating immunogenic VLPs [33]. This research used mammalian cells as an expression host for assembling the PiCV rCap-VLPs. The main advantage of using this expression system is its ability to execute a complete post-translational modification of the recombinant protein that is crucial for controlling the localization, stability, and conformation of proteins [34]. HEK-293 cell line, which is a well-established platform in bioprocessing for viruses and viral vectors production, was utilized in the experiments. The use of this cell for the construction of VLPs has already been previously reported [27]. Mammalian cells are increasingly being used to generate VLP-based vaccines as exemplified by porcine circovirus (PCV), porcine parvovirus (PPV), Lassa virus (LASV), Marburg virus (MARV), and Ebola virus (EBOV) VLPs [35]. Due to the ability of VLPs to trigger strong immune response and to induce antibody production, VLPs are considered to be a potential novel vaccine candidate [24,36].

The structural protein encoded by PiCV *cap* (C1) gene is called capsid protein (Cap). This protein is not only responsible for the capsid assembly, but it is also used as antigen for antibody detection during PiCV infections [37]. Aside from Cap being the fundamental part of the circoviral capsids, it also plays an intermediate role in the penetration of viral DNA within the nucleus of its host [38]. With no method to propagate PiCV in cell cultures, a suitable alternative method for the detection of PiCV-specific serum antibody is the expression of recombinant capsid protein [39]. A previous study performed on PCV2 showed that this viral capsid protein induces immune response, which includes specific antibodies and interferon gamma (IFN-γ) production [40]. Moreover, the study was also able to detect the immune responses that were seen in the appearance of antibodies between two and four weeks after the exposure of piglets to PCV2 or porcine circovirus capsid protein. Another study demonstrated the capacity of PiCV rCap to induce immune response in both naturally infected and uninfected pigeons; however, the rate of immune response differs depending on the severity of PiCV infection [1]. Cap expression level was further increased with the use of codon optimization, which is a prominent approach for increasing the expression of heterologous proteins in eukaryotic cells. Additionally, this technique is widely applied for both recombinant proteins and viral vector production [41]. Taking into consideration that an approved vaccine against PiCV is not yet available, other disease control strategies against the spread of PiCV infection can be utilized. Another study demonstrated a therapeutic approach in controlling the spread of PiCV infection by subcutaneous treatment of pigeons with PiIFN-α that resulted in the upregulation of *Mx*1 gene and IFN-γ, which proved the antiviral effect of PiIFN-α [15].

In this study, vaccination with VLPs demonstrated an increase in antibody production of immunized pigeons. Furthermore, the challenge test of experimentally infected pigeons showed no detectable copies of PiCV in spleen samples post-immunization, therefore proving that the VLP treatment can significantly reduce PiCV viral loads in the spleen. Similarly, VLP immunization in mice and guinea pigs was able to significantly induce specific antibody responses to PCV2 Cap [42]. Meanwhile, in another study [43], the protective immune responses of a VLP-based PCV2 vaccine enhanced by the incorporation of a truncated form of flagellin significantly reduced viral loads in lung samples from mice. PiCV viral loads in the spleens of naturally infected nonvaccinated pigeons were also reportedly higher than those of the naturally infected pigeons vaccinated with recombinant PiCV Cap vaccine formulation [1].

To further understand the immunogenic response to the PiCV rCap-VLPs treatment in this study, T-cell and cytokines were quantified. It was observed that the immune response caused by the PiCV rCap-VLPs significantly increased the proliferation of various T-cell and cytokines. T-cells are an important part of the immune system for the cell mediated immunity and the activation of immune cells, while cytokines are important as signaling molecules to regulate immunity [44]. Specifically, the VLP group displayed a T cell proliferation stimulation index that is twice higher and IFN-γ fold change that is thrice higher as compared with the control group. A similar study on PCV2 VLPs demonstrated that after the vaccination with VLP, the level of IFN-γ production was higher than with Cap vaccine [43]. Administration of VLPs enhanced the T helper type 1 (Th1), which promotes inflammatory responses through the secretion of cytokines such as gamma interferon (IFN-γ) that activate macrophages and provide help to CD8+ cytotoxic T cells [45]. Similarly, studies on the immunogenicity a PiCV rCap vaccine formulation have also reported increased synthesis of IFN- γ in uninfected vaccinated pigeons compared to the uninfected nonvaccinated controls all throughout the observation period post-vaccination [1,13]. A high level of IFN-γ is important because it is responsible for the adaptive immune cells, mainly antigen-specific T lymphocytes. Furthermore, this cytokine is also associated with innate immunity, notably with natural killer and natural killer T cells [42]. On the other hand, expression of TGF- β-2 in vaccinated and control groups was observed to have no significant statistical difference. However, it was also observed that TGF-β-2 decreased to 0.25-fold in the VLP group compared with the control group. TGF-β-2 plays an important role in the formation of blood vessels, the regulation of muscle tissue and body fat development, wound healing, and immune system functions [46]. TGF-β-2 is also responsible for regulating the cells from growing and dividing too rapidly; thus, it can also suppress the formation of tumors. In this experiment, immunization of PiCV rCap-VLPs in pigeons resulted in the suppressed expression of TGF-β-2. Lastly, the vaccinated and control groups were also observed to have no significant difference with respect to the expression of IL-8, a chemokine involved in early inflammation. In another vaccination study testing a PCV2 subunit vaccine containing Cap, it was similarly observed that IL-8 did not significantly differ between the vaccinated group and the control group prior to and at the early stage of infection. Nonvaccinated animals were also observed to have consistently low levels of IL-8, while the vaccinated group had increased IL-8 levels several weeks post-infection, which demonstrated that the innate immune response of the vaccinated group was more efficient compared to the nonvaccinated group. In the same study, animals with very low to no detected viral load in the blood also had higher IL-8 levels, while highly viremic animals had consistently low levels of IL-8 during infection [47]. 

Since prophylaxis against PiCV is not available, potential approaches were proposed for further development of the vaccine research. To summarize the results of this research, it was demonstrated that PiCV rCap-VLPs was successfully produced using a mammalian expression system. PiCV rCap-VLPs induced antibody titers in immunized pigeons and significantly reduced viral titers in experimentally infected pigeons. The constructed VLPs were proven to be an effective potential candidate vaccine against PiCV. Additional assessments and research on VLPs generated by mammalian expression system are required to further test the effectiveness of this vaccine.

## 5. Conclusions

The nonavailability of laboratory protocols for culturing pigeon circovirus (PiCV) and prophylaxis against the virus causes severe losses in the pigeon industry. A mammalian expression system was used in this study to obtain PiCV rCap-VLPs. Immunization with PiCV rCap-VLPs was shown to induce antibody response and significantly reduced the viral titer in infected pigeons, which proved its potential as a vaccine candidate. Further assessment of the VLPs generated by mammalian expression system is needed to investigate the efficacy of this potential prophylaxis. Results from this experiment provide important insights into the future development of PiCV vaccine research.

## Figures and Tables

**Table 1:** Primers used for quantitative real-time PCR.

Primer Name	Sequence	Size	Sequence ID	Template Position
PiCV	Forward: 5′ CTGACAGTGGGTCTCAACGC 3′	205 bp	GQ844278.1	217–236
	Reverse: 5′ CGTCAAAGTCCATGAGGGGG 3′			421–402
β-actin	Forward: 5′ TCCTTCTTGGGTATGGAATCTGT 3′	203 bp	XM_005504502.2	806–828
	Reverse: 5′ TTTCATTGTGCTGGGTGCCA 3′			991–972
IFN-γ	Forward: 5′ ATCCTGAGCCAGATTGTTTCCA 3′	144 bp	NM_001282845.1	211–232
	Reverse: 5′ GATCCTTGAGGTCTTGCAGC 3′			358–339
IL-8	Forward: 5′ GCCAGTGCATAGCCACTCAT 3′	172 bp	NM_001282837.1	98–117
	Reverse: 5′ GCATTTACAATCCGCTGGACC 3′			269–249
TGF-β2	Forward: 5′ TCACTTCCACTGTGCTCACC 3′	192 bp	EU737359.1	290–309
	Reverse: 5′ AGGTAAGTCCGAGCCCCATA 3′			481–462

## Data Availability

The data presented in this study are available within the article.
